# Editorial: Biological Hazards in Food

**DOI:** 10.3389/fmicb.2016.02154

**Published:** 2017-01-09

**Authors:** Maria Schirone, Pierina Visciano, Rosanna Tofalo, Giovanna Suzzi

**Affiliations:** Faculty of Bioscience and Technology for Food, Agriculture and Environment, University of TeramoTeramo, Italy

**Keywords:** food-borne pathogens, food safety, food spoilage, food management

## History and introduction

Food safety is one of the main objectives related to public health protection. It is expected to prevent, minimize or eliminate risks on different stages of the food chain and in the meantime maintain, provide, and distribute high-quality food to meet consumer demands. However, millions of cases of food-borne diseases occur every year worldwide (Martinović et al., 2016). Their global impact on health and food quality assurance is well-known even if the full health effects, the kinds of unsafe food, and the economic costs are often undervalued or miscalculated, as well as the outbreaks of food-borne diseases are often unrecognized, unreported, or not investigated. The globalization can lead to a widespread distribution of foods with the introduction of new pathogens strictly associated to a specific geographical area. Nowadays foods travel long distances to be consumed worldwide but only in developed countries consumers are aware of potential presence of food-borne pathogens and surveillance and analytical methods for their detection are really effective, while in developing countries the agents and sources of food-borne diseases are mostly unknown (Wang et al., 2016). In addition, the growing number of emerging pathogens, changes of virulence of known pathogens and appearance of antibiotic resistance has potentially exposed consumers to a major risk of illness and on the other hand food industry has been required to improve the strategies to struggle of these threats.

Food-borne diseases can be caused consuming food or water contaminated by pathogenic microorganisms such as bacteria and their toxins, fungi, viruses, and parasites. Food can be contaminated both at the source as raw material, and during food processing up to storage and distribution. Also people (infected persons or carriers of pathogens) and the environment (food contact surfaces and facilities) can spread microorganisms on raw or processed food.

## Food-borne pathogens and food spoilage

Among the major food-borne pathogens there are *Listeria monocytogenes, Campylobacter* spp., *Escherichia coli, Staphylococcus aureus*, and with respect to these microorganisms the most surveillance attention from both government agencies and food industry is recommended. These pathogens are different in terms of epidemiology, physiology, host association, and virulence, but for their overall persistence a great and continue monitoring is required. Outbreaks of listeriosis in humans are reported worldwide every year, also with a moderate percentage of fatal cases (Scallan et al., 2011). The potential pathogenic profile and virulence characterization were investigated in *L. monocytogenes* strains isolated from Chinese retail ready-to-eat food (Wu et al.). The genetic variation and phenotypic characteristics of *L. monocytogenes* isolates from retail raw foods (Chen et al.), as well as the growth potential of isolates from milkshakes, prepared from naturally contaminated ice-cream scoops linked to a listeriosis outbreak (Chen et al.) were studied and reported in this eBook. The methods for cultural detection, enumeration, and molecular identification of this pathogen in different foods were also exposed (Law et al.).

Human campylobacteriosis is one the most commonly reported food-borne diseases connected with the consumption of dairy and poultry products. *Campylobacter jejuni*, followed by *Campylobacter coli* and *Campylobacter lari* are the most common species associated with human infections. The high genome diversity and plasticity within the *Campylobacter* genus need of genotypic methods for outbreak investigations (Di Giannatale et al.).

*Escherichia coli* is a facultative anaerobic microorganism commonly found in human intestine but several strains have acquired virulence traits and can cause illness in humans. Among these strains *E. coli* O104:H4 has been associated with food-borne diseases recently occurred in some countries in the world. The development of an immune-magnetic separation method, by using of beads coated with monoclonal antibodies specific for the lipopolysaccharide of this pathogen from milk samples, was described in detail by Luciani et al.

Some original papers included in this research topic showed the prevalence of different pathogenic microorganisms such as *S. aureus* (Yang et al.) and *Vibrio parahaemolyticus* (Xie et al.) in a particular category of food (ready-to-eat) which do not need further processing before consumption and thus, the identification of microbial contamination is critical for assuring food safety. *Vibrio parahaemolyticus* has emerged as a major food-borne pathogen in China, Japan, Thailand, and other Asian countries. A total of 72 strains were isolated from clinical (i.e. patients with food poisoning) and food samples in China (Li et al.). The environmental parameters, strain sources and genotypes can affect the strain growth variability of *V. parahaemolyticus* and these researches can be helpful in predictive microbiology and microbial risk assessment (Liu et al.).

Another microorganism increasing importance as a food poisoning pathogen is *Bacillus cereus*. Some parameters (i.e., enterotoxin gene sequences, transcription, toxin secretion, and cytotoxicity) were studied in different *B. cereus* strains isolated from food and food poisoning outbreaks (Jeßberger et al.).

*Cronobacter sakazakii* is an opportunistic food-borne pathogen linked with life-threatening infections in infants (Song et al.). The virulent characterization (biofilm formation and flagella motility) studied by Ye et al. provided novel insides and better knowledge of the pathogenic mechanism of this microorganism.

Human noroviruses are major contributors to acute nonbacterial gastroenteritis outbreaks. A novel approach to characterize their interaction with receptors or ligands was explored by Niu et al.

If the consumption of unsafe foods can cause food-borne illness in consumers, microbial spoilage of food can be considered a greater issue than safety in terms of economic loss. It is estimated that food spoilage results in the loss of high rates of the total food supply (Pinu, 2016). Some acetic acid bacteria growing in wine can be considered spoilage bacteria because they metabolize ethanol to acetaldehyde by alcohol dehydrogenase and then produce acetic acid by acetaldehyde dehydrogenase (Longin et al.). Spoilage microorganisms such as *Lactobacillus buchneri* are able to produce acetic acid from lactic acid consumption under anaerobic conditions also in table olives, with consequent loss of their quality. In their review, Medina-Pradas and Arroyo-López reported other microbial metabolites of the product, which can also be considered toxic. Bacteria named macergens, which are responsible for plant tissue maceration by releasing pectic enzymes were described in the review of Aremu and Babalola.

## Other microbial toxins as public health hazards

Some chemical hazards are produced by biological agents and affect people with very different symptoms ranging from relatively mild discomfort to serious and life-threatening illness up to a fatal outcome. Among these hazards, marine biotoxins are produced by harmful algal blooms and can accumulate in bivalve molluscs. They are classified on the basis of their poisoning symptoms or their chemical structures. Regulatory limits and official detection methods have been established by the European legislation for these compounds (Visciano et al.).

The presence of biogenic amines in food and food products can be due to microorganisms (mainly bacteria) through the action of decarboxylases and environmental factors such as temperature, salt concentrations, and pH can influence their formation. Histamine and tyramine cause more severe acute effects in consumers. Some technological factors (i.e., addition of starter cultures, additives, effects of packaging) can be used for controlling their production (Gardini et al.).

Mycotoxin contamination in cereals and derived products represents an important problem for agriculture and food industries because it can cause substantial economic losses, mycotoxicoses in farmed animals and also toxic effects for human health. Aflatoxins are the most serious carcinogenic, hepatotoxic, teratogenic, and mutagenic secondary metabolites produced by fungi at any stage of food production from pre-harvest to storage. In recent years researchers reported new interventions to reduce or prevent the growth of fungi and subsequently mycotoxin production. The use of plant aqueous extracts was tested for their antifungal potential against aflatoxigenic isolates of *Aspergillus* spp. by Iram et al. and Iram et al.

## Food safety management systems

A food safety management system includes both control and assurance activities. Preventive measures aiming at avoiding contamination or outgrowth of microorganisms, as well as at their reduction or elimination, can be physical, chemical, and/or biological (Zwietering et al., 2016).

Equipment and food contact surfaces in food industries can provide a substrate for the development of the so-called biofilm, which represent a microbial community where bacteria can live in an extracellular matrix made of polysaccharides, extracellular DNA, and proteins. Cleaning and disinfection chemical products, such as surfactants and alkali compounds, can be used for the prevention of biofilm formation (Campana et al., 2017). Biofilm can be formed both on abiotic and biotic surfaces (Diaz et al.). The adherence of pathogenic microorganisms to surfaces and the formation of biofilm can be associated also to the production of biosurfactants. Rossi et al. reported that the biosurfactants produced by *Salmonella* Enteritidis SE86 contributed to adherence to slices of lettuce leaf and decreased the antimicrobial action of sanitizers used to sanitize whole lettuce leaves.

Some bacterial strains able to produce antagonistic molecules used as antimicrobials and preservatives can represent bio-control agents against harmful or pathogenic microorganisms in food. *Lactobacillus plantarum* is one of the most versatile species used in food industry as microbial starter or probiotic microorganism. Different strains of this species were shown to produce antimicrobial compounds and bacteriocins (Arena et al.).

Among physical approaches for food safety management, Patrignani and Lanciotti reported the application of high and ultra-high pressure homogenization for microbial inactivation and food safety purpose, even if they are not yet implemented in food industry, because they do not guarantee the sterilization of food.

## Conclusions

Food-borne diseases represent a significant problem for individuals, communities and food industries. Many factors are involved in these outbreaks such as a lacking quality of raw material, uncorrected handling of food, poor personal hygiene, and improper holding times/temperatures along the food chain. The articles included in this research topic range from food-borne pathogens and their metabolites/microbial toxins to preventive measures and management approaches to control and reduce/eliminate these hazards for the public health.

All the papers published within this eBook are reported in the references.

## Author contributions

MS and PV drafted the editorial, RT and GS contributed to editorial revision. All authors approved the final paper.

### Conflict of interest statement

The authors declare that the research was conducted in the absence of any commercial or financial relationships that could be construed as a potential conflict of interest.
